# Supplementary material for: De Novo p.Asp3368Gly Variant of Dystrophin Gene Associated with X-Linked Dilated Cardiomyopathy and Skeletal Myopathy: Clinical Features and In Silico Analysis
Source: Int J Mol Sci. 2024 Feb 28;25(5):2787. doi: 10.3390/ijms25052787 (PMC10931831; doi:10.3390/ijms25052787)
Supplement: Supplementary file 1 [file ijms-25-02787-s001.zip › ijms-2876524-supplementary.pdf]

S1. Gene list associated to Dilated Cardiomyopathy

RYR2, HFE, FHL1, SLC40A1, FHL2, PRKAG2, ILK, LDB3, TAZ, MPO, MYPN, SYNE2, SYNE1, CSRP3, SGCD, TMEM43, SGCB, HJV, PPP1R13L, LAMP2, NPPA, GATAD1, ANKRD1, EPG5, SCN5A, EMD, SCN1B, TMPO, MYBPC3, DSP, ACTN2, TNNC1, TPM1, ABCC9, SDHA, ACTA1, TFR2, MYL2, TNNT2, MYL3, PKP2, RBM20, DSG2, CRYAB, VCL, DSC2, MYH6, MYH7, CTF1, LAMA4, PSEN2, NEXN, PSEN1, TTN, PDLIM3, PLN, TTR, BAG3, PRDM16, LMNA, TNNI3, DMD, FLNC, NKX2-5, FKTN, DNAJC19, JUP, DMPK, NEBL, TXNRD2, IDH2, EYA4, CAVIN4, DOLK, XK, ACTC1, DES, RAB3GAP2, TCAP, HAMP, GLA.

S2: List of rare variant passed filtering strategy.

**Ref.Seq**	**SNP**	**GENE**	**cDNA variant**	**Protein alteration**	**Variant type**	**Prediction**
NM_182961.3	rs770713708	SYNE1	c.2543G>C	p.(Ser848Thr)	Missense	VUS
NM_182961.3		SYNE1	c.23020-3T		Splice region Intron	VUS
NM_004387.3	rs763099269	NKX2-5	c.335-297del		Intron	Benign
NM_198056.2	rs41258454	SCN5A	c.3511+10C>T		Intron	Likely Benign
NM_000116.4	rs113130344	TAZ	c.-88G>C		5-prime UTR	Likely Benign
NM_004415.3	rs142494121	DSP	c.2723G>A	Arg908His	Missense	Likely Benign
NM_000117.2	rs139983160	EMD	c.428C>T	p.(Ser143Phe)	Missense	Likely Benign
NM_182914.2	rs769211907	SYNE2	c.788-7C>G		Splice region Intron	Likely Benign
NM_182961.3	rs886044642	SYNE1	c.17203-5C>T		Splice region Intron	Benign
NM_182961.3	rs35379711	SYNE1	c.9489A>G	Gln3163=	Synonymous	Likely Benign
NM_002290.4	rs71543223	LAMA4	c.827_828delinsAC	p.(Asp276=)	Synonymous	Benign
NM_014000.2	rs71579355	VCL	c.1317T>C	p.(Ser439=)	Synonymous	Benign
NM_182961.3	rs141671123	SYNE1	c.6339T>C	p.(Thr2113=)	Synonymous	Likely Benign
